# Anterior cervical discectomy and fusion with plate versus posterior screw fixation after traumatic subaxial fractures in octogenarians: complications and outcomes with a 2-year follow-up

**DOI:** 10.1007/s00701-023-05566-x

**Published:** 2023-03-30

**Authors:** Pavlina Lenga, Gelo Gülec, Karl Kiening, Andreas W. Unterberg, Basem Ishak

**Affiliations:** Department of Neurosurgery, Heidelberg University Hospital, Heidelberg, Germany

**Keywords:** Subaxial fractures, Octogenarians, ACDF, Instrumentation

## Abstract

**Introduction:**

Surgical intervention for management of spinal instability after traumatic subaxial fractures in octogenarians requires a clear consensus on optimal treatments. This study aimed to provide a guide for more efficient management through comparison and assessment of clinical outcomes and complications of anterior cervical discectomy and fusion with plate (pACDF) and posterior decompression fusion (PDF) instrumentation alone in patients aged 80 years.

**Methods:**

A single institution retrospective review of electronic medical records was undertaken between September 2005 and December 2021. Comorbidities were assessed using the age-adjusted Charlson comorbidity index (CCI). Logistic regression was used to identify potential risk factors for ACDF complications.

**Results:**

The rate of comorbidities were similarly high between the pACDF (*n*=13) and PDF (*n*=15) groups (pACDF: 8.7 ± 2.4 points vs. 8.5 ± 2.3 points; *p*=0.555). Patients in the PDF group had significantly longer surgical duration (235 ± 58.4 min vs. 182.5 ± 32.1 min; *p*<0.001) and significantly higher volumes of intraoperative blood loss (661.5 ± 100.1 mL vs. 487.5 ± 92.1 mL; *p*<0.001). The in-hospital mortality was 7.7% for the pACDF group and 6.7% for the PDF group. On day 90, the mortality rate increased in both groups from baseline (pACDF: 15.4% vs. PDF: 13.3; *p*>0.05). Motor scores (MS) improved significantly after surgery in both groups (pACDF: preOP MS: 75.3 ± 11.1 vs. postOP MS: 82.4 ± 10.1; *p*<0.05; PDF: preOP MS: 80.7 ± 16.7 vs. postOP MS: 89.5 ± 12.1; *p*<0.05). Statistically significant predictors for postoperative complications included longer operative times (odds ratio 1.2, 95% confidence interval 1.1–2.1; *p*=0.005) and larger volume of blood loss (odds ratio 1.5, 95% confidence interval 1.2–2.2; *p*=0.003).

**Conclusions:**

Both pACDF and PDF can be considered safe treatment strategies for octogenarians with a poor baseline profile and subaxial fractures as they lead to patients substantial neurological improvements, and they are accompanied with low morbidity and mortality rates. Operation duration and intraoperative blood loss should be minimized to increase the degree of neurological recovery in octogenarian patients.

## Introduction

Currently, a steady global trend of increasing life expectancy is being observed [2], and the population of elderly people will approximately double by 2050. Aging can result not only in a rapid decline in mental and physical health, but also increase risk of disease or mortality. For example, spinal trauma with concomitant spine fractures presents a very common pathology in the elderly which causes falls from a standing height or sitting position and is associated with high morbidity and mortality [8, 14]. Cervical spine fractures disproportionally affect elderly patients [8, 16] with the axial spine appearing to be more prone to injury, although the prevalence of subaxial fractures is also considerable [25, 36]. In the case of spinal cord compression and spinal instability, surgical approaches such as posterior or anterior stabilization are recommended for the management of subaxial fractures [4, 29]. Particularly, an anterior approach is recommended for the presence of retropulsed bone fragments or injury of the disc, while posterior approaches, such as decompression and posterior screw fixation, are predominantly chosen for cases with posterior ligamentous disruption without dislocation or irreducible facets [29]. However, robust evidence on the optimal management of such fractures remains debatable. Several classification systems have been proposed for supporting spinal surgeons in decision-making [26, 29]. Notably, despite this dilemma of an anterior or posterior approach, the management of such fractures poses a unique challenge for older patients, especially octogenarians due to their high morbidity and mortality risk, attributable to a poor baseline reserve [33].

Most importantly, existing evidence reports mainly surgical procedures and outcomes on young patients, while the clinical course and decision-making for octogenarians are still marginal. In cases of octogenarians, spine surgeons are reluctant concerning whether to treat or not, mostly attributable to their poor baseline reserve. Potential risk factors, affecting mortality and prognosis in this population especially after the performance of surgery, are not available. Considering the steady increase of this population and their multitude of needs, there is an imperative need to elucidate the advantages and drawbacks of surgery for the treatment of subaxial fractures in this population.

**Fig. 1:**
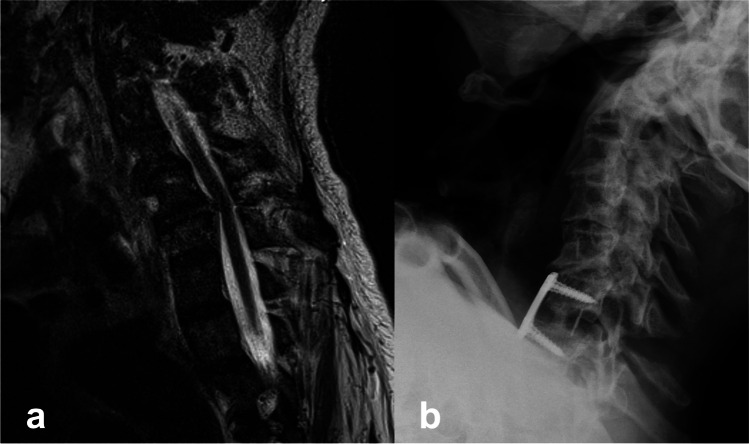
85-year female patient presenting with progressive motor weakness noted of the lower extremities. **a** Emergency MRI showing fracture at C5–C6 with bilateral facet dislocation and compression of the spinal cord. disruption. **b** Patient underwent fracture reduction and anterior cervical discectomy and fusion with plating

**Fig. 2:**
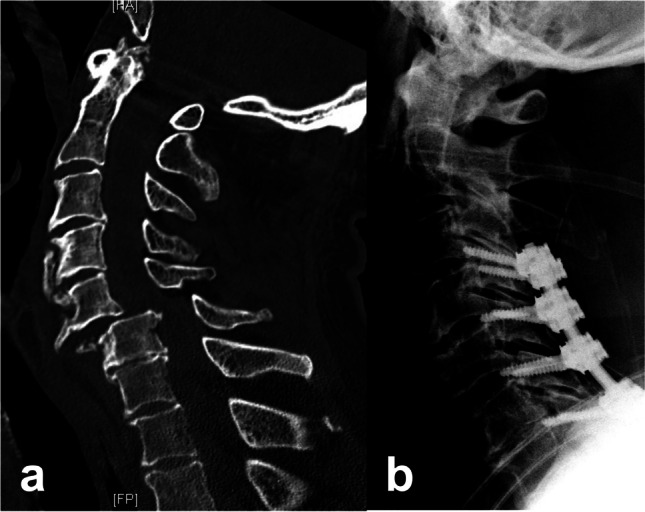
82-year male patient presenting with progressive motor weakness noted in the triceps, and poor hand response. His lower extremities were 1/5. **a** Emergency CT showing fracture dislocation at C5–C6 with anterior and posterior ligamentous disruption. **b** Patient underwent fracture reduction and posterior instrumentation from C4 to C7

Therefore, the aim of this study is to investigate and compare the clinical outcomes and mortality rates of anterior cervical discectomy and fusion (ACDF) with anterior plating or posterior fixation, with or without decompressive laminectomy in octogenarians who sustained subaxial fractures due to acute trauma Figs 1 and 2.

## Methods and materials

### Study design, inclusion, and exclusion criteria

This was a retrospective analysis of clinical and imaging data collected to the database of our institution between September 2005 and December 2020. This study was approved by the institutional ethics review committee (approval number 880/2021) and was conducted in accordance with the Declaration of Helsinki. The requirement for informed consent was waived due to the retrospective nature of the study. Our institution is a specialized spine trauma center. We exported all data regarding spinal subaxial fractures treated in our center regardless of the therapeutic procedure (surgery vs. conservative) from 2005 to 2020. We treated a total of 1345 patients (aged ≥18 years) with subaxial fractures. Of these cases, 28 patients (2.1%) were aged 80 years and older (range 80–90 years). After a meticulous study of the data, none of the patients aged more than 90 years with a subaxial fracture underwent a surgical approach. None of the patients aged 80–90 years underwent conservative managment.

Patients of 80 years old with traumatic subaxial fractures (C3–C7) diagnosed on computed tomography (CT) imaging were included. Magnetic resonance imaging (MRI) of the cervical spine was performed to evaluate spinal ligament integrity. The exclusion criteria were congenital instability, rheumatoid arthritis, instability caused by a tumor, spinal infections, and previous spinal surgery in the fracture region. The fractures were retrospectively scored according to the Subaxial Injury Classification (SLIC) system [38]. This classification system is based on fracture morphology, discoligamentous integrity, and neurological status. Preoperative radiological examinations were performed by an experienced neurosurgeon and a neuroradiologist. Both experts assigned SLIC points for morphological and discoligamentous injuries at the subaxial level. A score ≥ 4 points signified surgical recommendation. The German guidelines for trauma mechanisms were used to define low-energy trauma (LET) and patient injuries were classified accordingly [31]. LET was defined as a fall from a sitting or standing position at a low height (<1 m) [31].

### Surgery

Decision-making and discussion on the operative intervention was performed by an interdisciplinary team of neurosurgeons, neuroradiologists, and anesthesiologists in accordance with the clinical needs of each patient. All surgeries with instruments were performed using a CT-based point-to-point navigation system to ensure maximal safety, as previously described by our study group [17]. The final decision was determined by the senior spine surgeon. A broad spectrum of subaxial injury patterns exist and no consensus or scientific evidence provides definitive guidelines regarding the most effective surgical approach. Thus, in our department, the following techniques were used and patients were allocated into one of the two groups: (1) the anterior cervical discectomy and fusion with plate (pACDF) group or (2) posterior decompression and fusion (PDF) group. According to our institutional standards, patients with subaxial fractures received lateral mass fixation according to the Margel technique [21]. With Magerl’s technique, the center of the lateral mass was identified. The trajectory was angled at 45°–60° anterosuperiorly (parallel to the overlying facet joint) and 25° lateral to the sagittal plane. The screw length was typically 14 mm.

The basic goals of both procedures are adequate decompression of neural elements, restoration of alignment, and sufficient spinal stability.

According to the German Guidelines for the treatment of cervical fractures, a stiff collar after an anterior approach is not needed; thus, none of our patients received one post-surgery [29].

### Electronic records

Patient demographics, comorbidities, American Society of Anesthesiologists (ASA) scores, surgery duration, volume of blood loss, number of treated spinal levels, perioperative and postoperative complications, hospital length of stay, intensive care unit (ICU) stay, readmission rates, reoperation rates, and mortality rates were retrieved from patients’ electronic medical records. Comorbidities were preoperatively assessed using the age-adjusted Charlson comorbidity index (CCI) [9, 11]. The CCI score was calculated for each patient to classify comorbidities and was grouped as no comorbidity (CCI = 0), minimal comorbidity (CCI = 1 or 2), moderate comorbidity (CCI = 3–5), or severe comorbidity (CCI > 6). The preoperative neurological condition was assessed using the motor score (MS) of the American Spinal Injury Association impairment grading system (MS = 0, no muscle strength; MS = 100, healthy). Postoperative MS was documented according to the last clinical and imaging follow-up examination. According to our institutional standards, routine clinical and radiological follow-up examinations were performed before discharge and three months after surgery. The final follow-up was 26.7 ± 4.1 months postoperatively. Conventional radiographs in the anteroposterior and lateral views were obtained to evaluate the screw position and fusion rates.

### Statistical analysis

Categorical variables were presented as frequency counts and percentages. Continuous variables are presented as means ± standard deviations. They were normally distributed as verified using the Shapiro–Wilk test. Univariate analysis was used to compare groupwise baseline and surgical characteristics. For categorical variables, the chi-square test was applied. Independent *t*-tests were used for continuous variables. Potential risk factors for surgery complications were examined as independent variables using a binary logistic regression analysis model. The threshold for statistical significance was set at a *p*-value < 0.05. All statistical analyses were performed using SPSS software, version 24.0 (IBM Corp., Armonk, NY, USA).

## Results

### Patient demographics

Twenty-eight patients aged 80 years who were diagnosed with acute subaxial traumatic fracture requiring a surgical procedure were enrolled in the study. Thirteen patients were enrolled into the pACDF group and 15 were enrolled into the PDF group. Patients in the pACDF group were significantly older (mean age: 85.2 ± 1.5 years) than those in the PDF group (82.2 ± 1.1 years) (*p* = 0.015). The rates of comorbidities as measured by the CCI were similarly high in both groups (pACDF: 8.7 ± 2.4 points vs. 8.5 ± 2.3 points; *p*=0.555). The prevalence of chronic obstructive pulmonary disease was significantly higher in the PDF group (*p*<0.05). Patients in both groups presented similar neurological symptoms, according to MS. The baseline characteristics are depicted in Table 1.

**Table 1:** Baseline patient characteristics

	Anterior *n*=13	Posterior *n*=15	*p*-value
Age, mean (SD), years	85.2 (1.5)	82.2 (1.1)	**0.015**
Sex, *n* (%)			0.390
Male	9 (69.2)	8 (53.3)	
Female	4 (30.8)	7 (46.7)	
BMI, mean (SD), kg/m^2^	27.6 (4.7)	27.7 (6.5)	0.659
Comorbidities			
Age-adjusted CCI score, mean (SD)	8.7 (2.4)	8.5 (2.3)	0.555
Arterial hypertension, *n* (%)	13 (100.0)	10 (66.7)	0.322
Myocardial infarction, *n* (%)	6 (46.2)	6 (40.0)	0.743
Coronary heart disease, *n* (%)	8 (61.5)	6 (40.0)	0.256
Atrial fibrillation, *n* (%)	6 (46.2)	6 (40.0)	0.743
Peripheral vascular disease, *n* (%)	5 (38.5)	7 (46.7)	0.662
COPD, *n* (%)	0 (0.0)	6 (40.0)	**0.010**
Type 2 diabetes mellitus, *n* (%)	4 (30.8)	2 (13.3)	0.262
Renal failure, *n* (%)	1 (7.7)	5 (33.3)	0.099
Liver disease, *n* (%)	0 (0.0)	2 (13.3)	0.172
Gastrointestinal ulcer, *n* (%)	1 (7.7)	4 (26.7)	0.191
TIA/stroke, *n* (%)	2 (15.4)	2 (13.3)	0.877
Malignancy, *n* (%)	1 (7.7)	4 (26.7)	0.416
Dementia, *n* (%)	2 (15.4)	3 (20.0)	0.750
Previous spinal surgery	0 (0.0)	1 (6.7)	0.528
Active smoking, *n* (%)	1 (7.7)	1 (6.7)	0.290
ASA class, *n* (%)			0.823
II	3 (23.1)	4 (26.7)	
III	9 (69.2)	10 (66.7)	
IV	1 (7.7)	1 (6.7)	
Preoperative MS score, mean (SD)	75.3 (11.1)	80.7 (16.7)	0.088

*ASA*, American Society of Anesthesiology; *BMI*, body mass index; *CCI*, Charlson comorbidity index; *COPD*, chronic obstructive pulmonary disease; *TIA*, transient ischemic stroke; *MS*, motor score; *SD*, standard deviation

The *p*-values presented in bold font indicate statistically significant results

### Surgical characteristics, clinical course, and revision rates

As demonstrated in Table 2, patients in the PDF group had significantly longer surgical duration (235 ± 58.4 min vs. 182.5 ± 32.1 min; *p*<0.001), and significantly higher volumes of intraoperative blood loss (661.5 ± 100.1 mL vs. 87.5±18.1 ml mL; *p*<0.001). The lengths of ICU and hospital stay were similar in both groups. In-hospital mortality rates were 7.7% and 6.7% for the pACDF and PDF group, respectively. The 90-day mortality rate increased in both groups compared to baseline (pACDF: 15.4% vs. PDF: 13.3; *p*>0.05). Moreover, the postoperative neurological conditions and readmission rates did not differ between groups. Overall, the mean follow-up duration was 26.7 ± 4.1 months, and no secondary stabilization procedure was necessary. Furthermore, no screw loosening or displacement was observed on radiographs. The MS improved significantly postoperatively in both groups (pACDF: preOP MS: 75.3 ± 11.1 vs. postOP MS: 82.4 ± 10.1; *p*<0.05; PDF: preOP MS: 80.7 ± 16.7 vs. postOP MS: 89.5 ± 12.1; *p*<0.05).

**Table 2:** Comparison of surgical characteristics and clinical course between groups

Characteristic	Anterior *n*=13	Posterior *n*=15	*p*-value
Surgical duration, mean (SD), min	182.5 (32.1)	235.0 (58.4)	**0.021**
Segments, mean (SD	2.3 (1.4)	2.0 (1.0)	0.809
Surgical blood loss, mean (SD), mL	87.5±18.1	661.5 (100.1)	<0.001
Intraoperative blood transfusion, *n* (%)	3 (23.1)	4 (26.7)	**0.518**
Length of stay, *n* (%), days	10.0 (6.2)	11.3 (5.2)	0.204
ICU stay, mean (SD), days	4.9 (3.1)	1.9 (0.2)	0.089
Mortality			
In-hospital mortality, *n* (%)	1 (7.7)	1 (6.7)	0.455
90-day mortality, *n* (%)	2 (15.4)	2 (13.3)	0.457
30-day readmission, *n* (%)	0 (0.0)	0 (0.0)	-------
Post-MS, mean (SD)	82.4 (10.1)	89.5 (12.1)	0.098

*ICU*, intensive care unit; *MS*, motor score; *SD*, standard deviation

The *p*-values presented in bold font indicate statistically significant results

### Conservative management

Only three patients aged 92, 93, and 98 underwent conservative management with stiff neck collars. All patients presented with new neurological weakness and significant loss of strength in the low extremities. CT imaging revealed unstable subaxial fractures warranting the performance of posterior instrumentation and fusion. All patients suffered from multiple cardiovascular diseases, chronic renal failure, type II diabetes mellitus, and severe dementia. However, considering that patients will and after a clear discussion of the related risks with their family and relatives, we opted them for conservative management.

### Occurrence of adverse events and potential risk factors

No significant difference was observed between groups in regards to postoperative complications. A detailed breakdown of all recorded complications is presented in Table 3. Logistic regression analysis adjusting for relevant risk factors revealed a significant association between the occurrence of postoperative complications and longer operative times (odds ratio 1.2, 95% confidence interval 1.1–2.1; *p*=0.005) and with larger volume of 100 ml blood loss (odds ratio 1.5, 95% confidence interval 1.2–2.2; *p*=0.003), but not with age, sex, CCI, neurological status, surgical techniques, or number of operated segments (Table 4).

**Table 3:** Occurrence of adverse events and revision rates

	Anterior *n*=13	Posterior *n*=15	*p*-value
Superficial wound infection, *n* (%)	0 (0.0)	1 (6.7)	0.915
Acute respiratory failure, *n* (%)	1 (7.7)	1 (6.7)	0.575
Acute heart failure, *n* (%)	1 (7.7)	2 (13.3)	0.625
Thrombotic event, *n* (%)	1 (7.7)	0 (0.0)	0.915
Pneumonia, *n* (%)	3 (23.1)	4 (26.7)	0.295
Pleural effusion, *n* (%)	4 (30.8)	5 (33.3)	0.885
Urinary tract infection, *n* (%)	1 (7.7)	0 (0.0)	0.274

**Table 4:** Risk factors for complications

Complications	OR (95% CI)	*p*-value
Age	1.1 (0.8–1.3)	0.436
Sex^a^	5.8 (2.1-7.2)	0.444
Age-adjusted CCI score	1.2 (0.6–2.5)	0.965
MS	1.0 (0.9–1.1)	0.576
Fusion surgery^b^	12.1 (5.4–15.4)	0.290
Operated segments	7.1 (2.2–9.0)	0.160
Duration of surgery	1.2 (1.1–2.1)	**0.005**
Blood loss >100 ml	1.5 (1.2–2.2)	**0.003**

*OR*, odds ratio; *CI*, confidence interval; *CCI*, Charlson comorbidity index

^a^Reference, male sex

^b^Reference, anterior cervical discectomy and fusion with plate

The *p*-values presented in bold font indicate statistically significant results

## Discussion

To the best of our knowledge, this is the first study examining surgical strategies (pACDF vs. PDF) in octogenarians sustaining subaxial fractures after acute spinal trauma. The key result of the present study is that surgery (both anterior and posterior) has a good safety profile, with low mortality rates without the need for revision surgery due to secondary instability, and significantly improves the patients' neurological status.

Patients from both cohorts had similar neurological conditions, with a mean MS ranging between 74.3 and 80 points, indicating a high degree of disability. Interestingly, patients who underwent PDF had significantly longer duration of surgery with greater intraoperative blood loss than patients who underwent pACDF. However, these phenomena did not significantly affect the length of hospital stay or mortality rate. Reassuringly, both surgical techniques led to significant improvements in motor weakness. The unique risk factors for the occurrence of complications were duration of surgery and blood loss, whereas the surgical technique itself was not. Revision surgery or readmission was not necessary in the 2-year follow-up period irrespective of the surgical approach used.

Age is a known risk factor for increased morbidity and mortality, producing reluctance of spine surgeons to perform surgical procedures on the older population due to potential complications or occurrence of death [5, 35]. Tan et al. retrospectively analyzed 47 octogenarians undergoing spine surgery with the aim of determining potential risk factors for morbidity and mortality. They stated that both the presence of multiple comorbidities and vertebral fractures (pathologic and traumatic) were associated with higher mortality rates, while elective or emergency surgery, even in cervical segments, was not [35]. In conjunction with these findings, based on data from 1789 older patients undergoing cervical spine surgery due to different cervical pathologies, Bernstein et al. also concluded that higher rates of underlying diseases (as defined by the ASA score) and age > 75 years are significant risk factors for the occurrence of complications or even death within the first 30 days after surgery [5]. In another study on cervical spine injury comparing patients of different age ranges, the authors assumed that the occurrence of complications such as cardiac or respiratory decompensation might be attributable to the poor baseline history of older patients, thus affirming the urgent need for a meticulous investigation of this subset of patients with a sufficient counterbalance of pros and drawbacks of a surgical procedure [33]. Tarawneh et al. also advocated that older patients with higher rates of underlying diseases who sustained cervical fractures are at higher risk for mortality because of the substantial decrease in their functional status and, most importantly, the ability to cope with surgery-related complications and infections. As a result, poor prognosis is expected compared to younger counterparts [36]. In the present study, octogenarians suffering from acute traumatic subaxial fractures were debilitated before surgery due to their poor baseline history, as indicated by the CCI. Furthermore, we did show that patients undergoing PDF suffered more frequently from COPD, which might explain why this subset of patients presented more often with postoperative pneumonia. It is well known that patients with COPD are more likely to have severe pneumonia and are linked to worse outcomes. Previous studies have stated that patients with COPD are more amenable to even a 4-fold risk of developing pneumonia compared to healthy counterparts [18, 27], which might also be attributable to the use of corticosteroids. Considering these findings, such patients should be closely monitored after surgery to avoid the development of such complications.

Notably, the comorbidity rates did not constitute a significant risk factor for the presence of comorbidities as was previously shown. In light of these findings, we strongly believe that while the unique needs of patients in this cohort present limitations for surgery, they should be carefully factored in when selecting the safest and most optimal surgery.

Of note, longer surgery times and intraoperative blood loss of at least 500 mL were significant predictors for the occurrence of postoperative complications, irrespective of the treatment approach (anterior vs. posterior fusion) or baseline clinical and neurological status. Similarly, Bernstein et al. analyzed patients aged ≥ 65 years with cervical injury and concomitant fracture who underwent surgery and stated that an operation time longer than 180 min was significantly associated with the presence of postoperative complications, irrespective of whether an anterior or posterior approach was undertaken [5]. Nevertheless, patients undergoing ACDF were at an increased risk of complications in the presence of at least one comorbidity or progressive neurological decline, while patients undergoing decompression surgery were not [5]. Likewise, a study based on claims data from 58,111 patients with cervical spondylotic myelopathy reported the association of longer operation time and greater complication rates, while posterior fusion led to significantly higher complication rates when compared to an anterior approach [6]. In contrast, in the retrospective study by Schoenfeld et al., 3475 patients with spinal pathologies who underwent spine surgery reported that longer operation time was a significant risk factor for the occurrence of postoperative complications, while the surgical technique itself was not a risk factor [30]. It should be noted that unlike the aforementioned studies, this study focused on octogenarians with acute traumatic subaxial fractures who underwent surgery. The surgical approach in this study was individualized after meticulous examination of both clinical and imaging characteristics in an interdisciplinary concept, aiming to select the most suitable therapeutic strategy for such a frail cohort. Another important aspect is that placement of lateral mass screw fixation was performed with point-to-point intraoperative CT navigation, thus minimizing perioperative pedicle screw misplacement.

Octogenarians undergoing surgery for subaxial fractures have a high risk for complications and mortality. Bernstein et al. reported that even those aged age ≥70 years presented with at least one postoperative complication after studying a large cohort of 1786 patients with cervical pathologies [5]. Pneumonia, urinary tract infection, myocardial infarction, and deep vein thrombosis were significant predictors for the occurrence of postoperative complications in patients aged ≥ 80 years [5]. Similarly, another study analyzing complication and mortality rates after cervical spinal fusion in patients with different cervical pathologies and traumatic subaxial factors found that geriatric patients had an increased risk of postoperative adverse events (pneumonia, deep vein thrombosis, and urinary tract infection) and even death [10]. The retrospective study by Fredo et al. on 303 patients (age range: 15–94 years) who sustained acute traumatic subaxial fractures reported mortality rates of 2.3% in the first 30 days post-surgery, irrespective of surgical procedure (anterior vs. posterior approach), with older patients having an increased risk of death due to postoperative complications such as pneumonia or concomitant injuries such as brain injury [15]. Daneshvar et al. retrospectively analyzed 37 octogenerians with acute cervical fractures and reported a 38% in-hospital mortality rate without surgical procedure [8]. Aside from patient age and comorbidities, another risk factor was trauma severity resulting in respiratory failure. In contrast, according to our results the overall in-hospital mortality rate post-surgery was significantly lower (by 7.7%) after ACDF with plate, and 6.7% lower after decompression and posterior screw fixation. Interestingly, the cause of death was not incurred due to surgery. One patient experienced septic pneumonia after ACDF and one died after posterior screw fixation due to acute heart failure. Most importantly, no significant differences in mortality rates were observed with respect to the surgical approach used and no revision surgery due to implant failure or screw loosening was necessary. It should be emphasized that these patients were admitted to the ICU or an intermediate care ward for thorough monitoring after surgery. Therefore, close monitoring of frail patients in the ICU may have permitted early diagnosis and better management of any potential complications. Moreover, previous studies have reported that elderly patients (> 80 years) undergoing surgery might benefit from ICU care, although prompt transfer to the regular ward should be ensured to limit the development of new or additional disability affecting activities of daily living, which can lead to poor long-term outcomes [24]. Of note, ICU utilization is associated with high cost and demand for resources. Such care is more feasible in Western countries; hospitals in rural areas lack such facilities, and thus the mortality rates of octogenarians might be higher in such areas [23]. At day-90, mortality rates substantially increased compared to baseline. This might be attributable to the fact that octogenarians are already debilitated due to their poor baseline health. Hence, these patients are more amenable to complications, such as infection or cardiovascular diseases. Irrespective of the treatment, cause of death was cardiovascular disease or respiratory failure, not primarily surgery-related. Therefore, we believe that our findings indicate that even in octogenarians suffering from such acute fractures with devastating consequences, surgery (anterior plating or posterior screw fixation) can contribute to the mitigation of mortality rates if patients are left untreated [8].

There remain still some controversy regarding the use of collars before and after surgery to subaxial fractures. In a retrospective study on 74 young patients (mean age, 55 years) with nondisplaced subaxial fractures, Van Eck et al. reported treatment failure due to olisthesis in almost 11% of the examined cases; thus, surgery was needed in more than 10% of cases [13]. In contrast, Aarabi et al. found higher failure rates reaching almost 60% with non-surgical management of nondisplaced subaxial. This was mainly attributable to the enrolled patients’ young age and subsequently a lower compliance to wearing a rigid collar [1]. In line with these findings, Spector et al. also reported limited successful treatment rates of immobilization with a rigid collar in nondisplaced subaxial fractures with progressive dislocation in 21% of cases [34]. In another review and meta-analysis of the merit of collars for subaxial non-subluxated facet fractures, the study group concluded that surgical management seems to be the gold standard even for nondisplaced fractures, and that the presence of neurological deficits or even a slight ligamentous involvement is a critical prognostic factor for conservative treatment failure [20]. However, stiff collars may benefit in patients undergoing only an anterior or posterior procedure, whereas a combined procedure would have been optimal. Ren et al. in his retrospective study on subaxial fractures in 159 patients (mean age of approximately. 50 years) undergoing an anterior or posterior approach. They described using of postoperative collars for 12 weeks to protect for the cervical vertebrae. However, the authors could not substantiate the potential benefits of the postoperative collar use [28]. Moreover, Fredo et al. also used rigid collars on patients undergoing a single-stage surgery (anterior or posterior procedure); however the potential impact on further stability after surgery was not explicitly studied and could not be objectively proved [15]. Overall, the potential benefits of the postoperative use of rigid collars in subaxial fracture cases may require a combined approach. Their deployment mainly depends on the surgeons’ personal experience and the institution’s guidelines. In the case of older patients, as shown in this study, we believe that their use is further limited because of the low compliance of this age group. Since instability signs were not present and owing to inconclusive clinical evidence, we decided not to use a collar, which could have resulted in complications such as respiratory difficulties, wound infection, and dysphagia.

It is well known that a clear consensus for the management of subaxial fractures among surgeons is still marginal. Moreover, decision-making is mainly based on the surgeons’ experience and patient clinical condition. However, in certain types of fractures, such as vertebral burst fractures with the concomitant disruption of the posterior ligaments and injury of the facet capsule, a combined approach (anterior cervical discectomy and reduction and posterior lateral mass fixation and fusion) is recommended as the mainstay of treatment [3, 12, 19]. Such injuries are often related to severe or complete neurological injury; thus, an emergency anterior decompression of the dural sac and the nerve roots is warranted. Considering the disruption of the ligaments and the destruction of the vertebra, a combined approach seems to offer sufficient decompression and reconstruction of a stable spine [7, 12]. For example, in Miao et al.’s retrospective study, 24 patients with distraction/flexion injuries of the subaxial spine underwent a combined approach, leading to immediate neurological improvement of more than 50.0% after a 3-year follow-up [22]. In addition, bone fusion and an acceptable reconstruction of the spinal alignment were present within 4 to 6 months after surgery. It is important to highlight that their cohort consisted of young patients aged 21 to 65 years, thus, indicating a healthy baseline history [22]. Notably, surgical duration reached a mean time of 330 min, while the volume of blood loss was over 700 ml [22]. In line with these findings, Shen et al. also described that a combined approach is profitable and safe for this kind of fracture, but once again, their patients were younger (mean age of approximately 30 years) [32]. In contrast, Toh et al. advocated that anterior fusion can produce similar good outcomes with significant improvement of the neurological impairment compared to the combined approach and should be preferred when possible [37]. On the other hand, Dvorak et al. stated that a combined approach is recommended since anterior fusion achieves a sufficient anterior decompression and the integrity of the discoligamentous soft tissue complex can be preserved due to posterior and anterior fixation [12].

Considering all the aforementioned studies, there is the notion that a combined approach should be performed in such cases with this severe fracture pattern. However, as already emphasized, previous studies focused on younger patients. Thus, the surgical duration, blood loss, and general anesthesia did not lead to severe peri- and postoperative complications or even death. In the present study, only patients aged 80 years and older with a poor baseline reserve of 8.6 were analyzed; thus, the conduction of such an approach is presumably linked to major complications or even death. We did show that surgical duration and volume of blood loss are significant predictors of the occurrence of complications. In our institution, every case was meticulously studied by experienced spinal surgeons (BI, KK), neuroradiologists, and anesthesiologists. Therefore, the less risky and beneficial treatment approach for the reconstruction of spinal stability was chosen. A combined approach was not performed due to the overdue risks for this subset of patients. The anterior fusion with plating also achieved preservation of spinal alignment, sufficient decompression, and stabilization of the spine. No revision surgeries were performed due to secondary instability. Most importantly, patients’ neurological impairment improved substantially, as defined by the MS score. Therefore, we feel that in such a frail cohort, an anterior approach might lead to similar outcomes as a combined one since these patients, respective to their baseline histories, are at higher risks of morbidity and mortality.

Acute traumatic subaxial fractures are associated with poor functional outcomes, especially in elderly patients, and the presence of osteoporotic bones reduced recovery capacity, and medical comorbidities predispose octogenarians to higher risks of negative outcomes. Notwithstanding, our findings highlight that even this subset of patients recovered successfully after surgery with significant improvements in functional status, as indicated by the MS score. Most importantly, an improvement in neurological status was observed, irrespective of the surgical procedure. However, determining the optimal treatment for such patients is difficult, and decision-making relies on many factors such as the surgeon in training, patient-specific factors, and personal preference. In their review of literature and meta-analysis, Patel et al. examined different classification systems for the management of subaxial fractures with the aim to determine a treatment algorithm that would be a useful tool for physicians [26]. The classification system proposed by Dvorak et al. seems sufficient for describing such fractures [12]. According to the proposed scale, the 3 components of injury (morphology, neurology, and discoligamentous soft tissue complex disruption), which, by consensus, represent major and largely independent determinants of prognosis and management, is an attempt to characterize injuries based on morphology and clinical status. For instance, anterior decompression and fusion is recommended for burst fractures or compression and distraction injuries, while hyperextension injuries with concomitant spondylotic cervical spine should be fixated posteriorly. In the present study, we closely evaluated the type of fractures with an interdisciplinary team of experienced neuroradiologists and then after meticulous examination of the unique needs of each patient, considering the high rates of comorbidities, we decided the surgery that provides both cervical stability as well as short operation times aiming to diminish intra- and postoperative complications. Since both neurological statuses significantly improved and no further surgery due to implant failure or secondary instability was needed, we believe that surgery, even in octogenarians, might be the treatment of choice that preserves neurological status while simultaneously reducing mortality rates.

### Strengths and limitations

The main strength of our study is that, to the best of our knowledge, this is the first report to examine solely octogenarians undergoing surgery for subaxial fractures. However, this study has some limitations. Selection bias could not be ruled out because of the retrospective study design. A relatively small cohort of patients was examined. Nevertheless, we believe that our findings give a broader clinical picture since there is a lack of robust evidence on the clinical course of such a devastating disease among older individuals. Moreover, as the data originated from a high-volume center, potential performance bias should also be considered. Longer follow-up periods may be necessary to uncover relevant information not captured in the current study. A selection bias might be present in the selection of the treatment approach. Notwithstanding, it is paramount to outline that there are still no guidelines or general consensus on how to treat such fractures. The final decision-making is mainly based on the discretion of the treating physician. In 2007, Dvorak et al. published an evidence-based algorithm based on fracture morphology to define the most suitable surgical approach for each fracture. The authors concluded that burst or compression fractures are more likely to be treated by an anterior approach, while translation or rotation injuries can be treated by a posterior or combined approach [12]. However, this algorithm is limited because it is based on a review of the literature (case reports and retrospective studies), the expert’s experience, and the inclusion of patient preferences based on informed probabilities. Although that might be an additional tool supporting decision-making, the final decision should be based on the patient’s characteristics, especially regarding octogenarians. Therefore, the presence of bias was also unavoidable in our cases.

Larger studies are warranted to better elucidate the multitude of needs of the octogenarian cohort. A smal number of patients was examined in the present study; thus limiting the statistical power of our findings. Herein, it should be emphasized that evidence concerning this age group has been marginal so far in the literature, a phenomenon mainly attribuatble to the reluctance for surgical managment owing to the high frailty rates and presumably the accompanying postoperative high morbidity and mortality rates. As expected, we did show that patients below 84 years may be more suitable for a posterior approach rather than those above 84 years; however, these findings need to be further substantiated in a prospective setting with a large number of patients. Nevertheless, the development of such a study design is difficult because of factors such as patient consent, recruitment, and emergency settings. Therefore, these findings produced by studying 28 patients showed that surgical management of subaxial fractures in this age group is associated with sufficiently good clinical outcomes and improved neurological status.

## Conclusion

Spine surgeons frequently encounter older patients requiring surgical therapy because of the steadily increasing average life expectancy. Our results show that both ACDF with plate and posterior decompression with instrumentation can be considered safe treatment strategies for patients with subaxial fractures since neurological improvements and low mortality rates were noted. However, the duration of surgery and blood loss should be minimized to avoid devastating complications. The potential benefits and risks should be unambiguously clear and discussed between patients and relatives for a mutual decision on the most optimal treatment plan.

## Data Availability

The datasets generated during and/or analyzed during the current study are available from the corresponding author on reasonable request.
